# Two Alkaloids From *Delphinium brunonianum* Royle, Their Anti-inflammatory and Anti-oxidative Stress Activity *via* NF-κB Signaling Pathway

**DOI:** 10.3389/fnut.2021.826957

**Published:** 2022-01-20

**Authors:** Qi Tang, Sitan Chen, Syed Arif Hussain Rizvi, Jiaojiao Qu, Li Wang, Senye Wang, Changyang Ma, Lijun Liu, Wenyi Kang

**Affiliations:** ^1^National R&D Center for Edible Fungus Processing Technology, Henan University, Kaifeng, China; ^2^Pakistan Agricultural Research Council, Islamabad, Pakistan; ^3^Functional Food Engineering Technology Research Center, Kaifeng, China; ^4^Joint International Research Laboratory of Food and Medicine Resource Function, Kaifeng, China; ^5^Huaihe Hospital, Henan University, Kaifeng, China

**Keywords:** *Delphinium brunonianum* Royle, anti-inflammatory, RAW264.7 cells, LPS, Delbrunine, Eldeline

## Abstract

In this study, we isolated and identified four compounds in *Delphinium brunonianum* Royle, and they were Delbrunine (**1**), 4-*O*-α-D-Glucosyl benzoic acid (**2**), Kaempferol 3-*O*-β-D-glucopyranoside 7-*O*-α-L-rhamnopyranoside (**3**) and Eldeline (**4**). Furthermore, the anti-inflammatory activity of these compounds was screened in RAW264.7 cells. The results showed that the anti-inflammatory activities of compounds **2** and **3** were weak, and **1**, **4** had good anti-inflammatory activity. The macrophage inflammation model was established by lipopolysaccharide (LPS). Then, the anti-inflammatory activity was evaluated by ELISA kits, qRT-PCR experiment and western blot experiment. And the anti-oxidative stress activity was assessed by flow cytometry. The results showed that compounds **1**, **4** could significantly inhibit the elevation of inflammatory factors nitric oxide (NO), tumor necrosis factor-α (TNF-α), interleukin-6 (IL-6), and also had obvious inhibitory effects on the production of inducible nitric oxide synthase (iNOS) and cyclooxygenase 2 (COX-2). In addition, compounds **1** and **4** could effectively inhibit the overexpression of reactive oxygen species (ROS) in RAW264.7 cells that activated by LPS. These results indicated that compounds **1** and **4** may exert anti-inflammatory and anti-oxidative stress effects through the NF-κB signaling pathway.

**Graphical Abstract:**
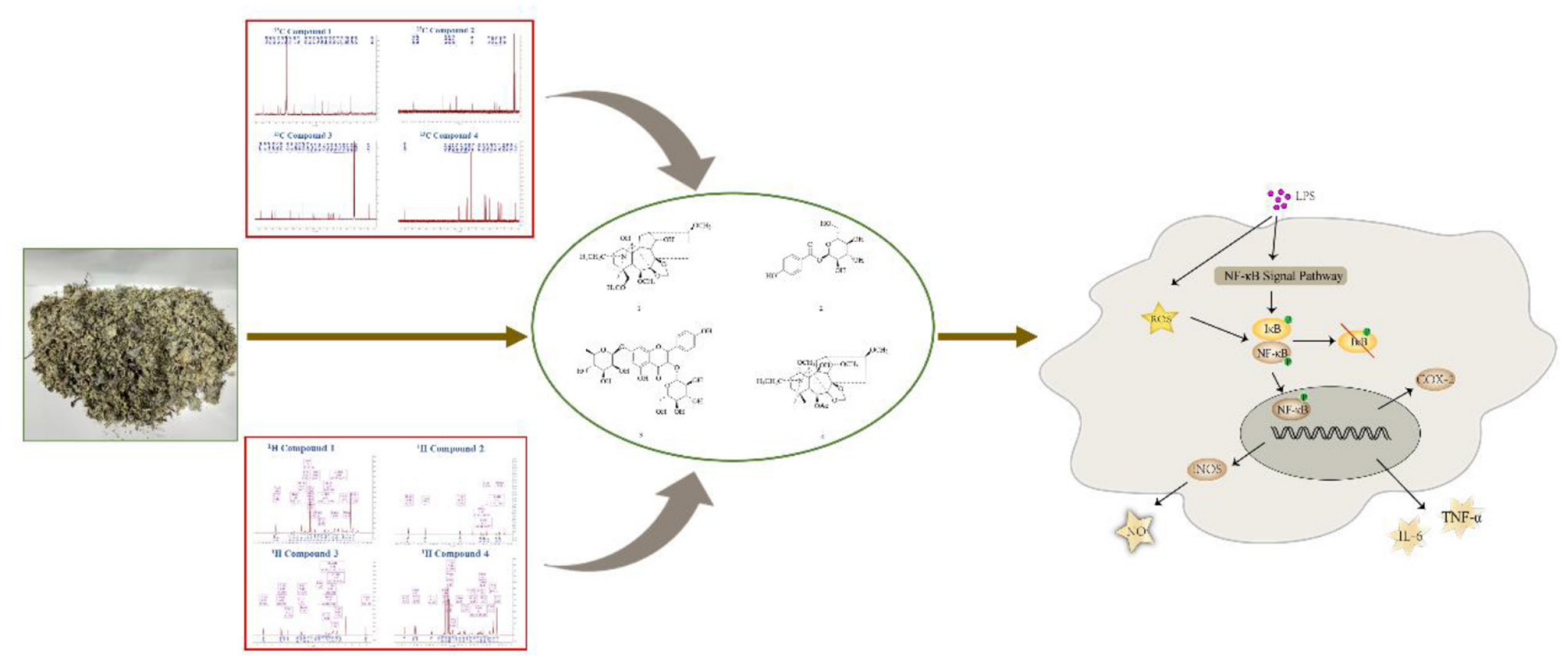
Two alkaloids with anti-inflammatory from *D. brunonianum via* NF-κB signaling pathway.

## Introduction

Inflammation is the basis of many pathological processes (1). The main symptoms associated with inflammatory disorders include body pain, arthralgia, myalgia, fever, local swelling and frequent infections (2). Infection and tissue damage are the main causes of inflammation, they recruit leukocytes and hemoglobin to transfer to the affected tissues (3). The metabolic diseases can also induce chronic inflammation in the body, such as insulin resistance, type 2 diabetes, coronary artery disease and fatty liver disease (4–6). During inflammation, several types of immune cells are activated, including macrophages, which have been found to play a central role in the anti-inflammatory process (7). The two basic functions of macrophages are phagocytosis and adaptive immune response (8, 9). Phagocytosis plays a vital in the body's response to acute inflammation, it can quickly resist the invasion of foreign pathogens. In addition, in chronic inflammation, it is also particularly important to remove apoptotic cells. And macrophages can present antigens together with dendritic cells (9–11). At the same time, macrophages can also secrete a variety of powerful catalytic factors, such as tumor necrosis factors and interleukins (12). Excessive release of inflammatory mediators can cause damage to the body, so inhibiting the production of these cytokines may be an effective strategy for the treatment of inflammatory diseases. NO is produced by L-arginine under the action of nitric oxide synthase. The expression of iNOS increases under inflammation and immune stimulation, thereby promoting the production of NO (13). Excessive NO could promote the occurrence and development of inflammatory diseases. In addition, TNF-α and IL-6 are the main cytokines that mediate inflammation. TNF-α is mainly produced by macrophages which can activate white blood cells and endothelial cells, causing extensive damage to tissues. IL-6 is the most important inflammatory mediator involved in inducing the acute phase protein response during fever (14).

**Figure 1:**
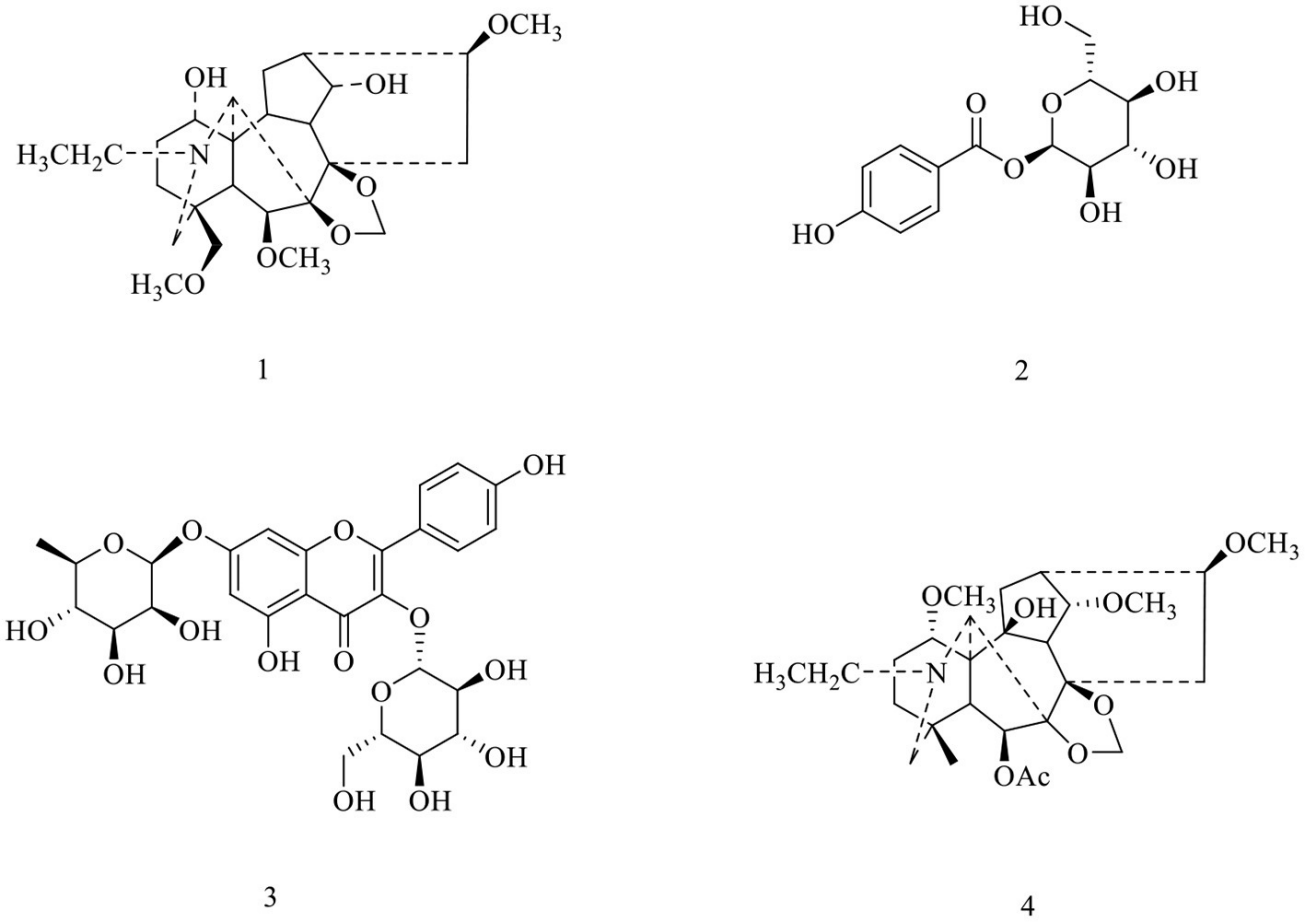
Structures of compounds **1**–**4** from *D. brunonianum*.

Although many synthetic anti-inflammatory drugs such as steroids, non-steroidal anti-inflammatory drugs and immunosuppressants have been widely used in inflammatory diseases, their long-term use is limited by related side effects (15, 16). Therefore, many plants are used to explore safe, effective and easily available anti-inflammatory drugs (17). Many studies have shown that medicinal plants can be used to treat inflammation and have been successfully converted into convenient and effective dosage forms for modernmedicine (18). *D. brunonianum*, belonging to *Delphinium* genus (Ranunculaceae family), usually has the effects of clearing heat, detoxifying, anti-inflammatory and analgesic as folk medicine. The past research showed that the alkaloids are the main constituents, and flavonoids and sterols were also found, with the effects of antibacterial, antiepileptic, detoxification, and Alzheimer's disease treatment. For example, hydroalcoholic extract and fractions obtained from *D. brunonianum* presented significant diuretic and natriuretic effects when given to rats through the oral route (19). Also, past research has shown that the anti-tumor activity of *D. brunonianum*, but its anti-inflammatory active ingredients and mechanism are still unclear (20). In this research, four compounds were isolated from *D. brunonianum*, identified as Delbrunine (21, 22), 4-*O*-α-D-Glucosyl benzoic acid (23, 24), Kaempferol 3-*O*-β-*D*-glucopyranoside 7-*O*-α-*L*-rhamnopyranoside (25) and Eldeline (26) by comparing with the published data (Figure 1). The purity of all compounds was more than 95%. Studies have shown that LPS can stimulate macrophages to induce inflammation and increase the production of reactive oxygen species (27). Therefore, this study established macrophage inflammation model using LPS to explore the anti-inflammatory activity and mechanism of *D. brunonianum*.

## Materials and Methods

### Reagents and Instruments

The following reagents were used in this research: RAW264.7 cells were purchased from the Typical Culture Preservation Committee Cell Bank, Chinese Academy of Sciences. DMEM (Solarbio, Beijing, China). FBS (Gibco, GrandIsland, USA). Penicillin-streptomycin mixture, Nucleoprotein extraction kit, BCA protein quantitative kit and protease phosphatase inhibitor (SolarBio, Beijing, China). LPS (Sigma-Aldrich, St. Louis, MO, USA). NO kit and ROS kit (Beyotime Biotechnology Shanghai, China). Mouse TNF-α Enzyme-linked immunosorbent assay (ELISA) kit and mouse IL-6 ELISA kit (4A Biotech Co, Ltd. Beijing, China). *Evo M-MLV* RT Kit with gDNA Clean and SYBG Green Premix *Pro Taq* HS qPCR kit (Tli RNadeH Plus, Accurate Biotechnology, Hunan, China). Antibody iNOS, COX-2, NF-κB P65, p-NF-κB P65 (Cell Signaling, USA). Primers were designed and synthesized by Thermo Fisher Scientific (Shanghai, China). The primers were shown in Supplementary Table 1.

### Extraction and Isolation

The dried aerial parts of *D. brunonianum* were extracted with petroleum ether 3 times, and the filter residue was extracted with 70% ethanol 3 times (each time for 7 days). By concentrating the combined extract under reduced pressure, 470 g of the extract was obtained. The extract was eluted by D101 macroporous resin with six solvents, which were water, 20% ethanol, 40% ethanol, 60% ethanol, 80% ethanol and 95% ethanol. Six components were obtained (Fr. A-Fr. F). Forty percent components (Fr. B 60 g) were separated by silica gel column chromatography, and 10 fractions fr.1-10 were obtained by gradient elution with CHCl_3_-MeOH (40:1-1:1).

Fr.1 was eluted by Sephadex LH-20 with methanol, then eluted with CHCl_3_-MeOH (40:1-1:1) gradient by silica gel column chromatography. Fr.1-3 was eluted with CH_2_Cl_2_-MeOH (50:1-1:1, v/v) gradient through reduced pressure silica gel column chromatography to obtain compound **1** (40 mg). Fr.5 was eluted with EtOAc-MeOH (40:1-1:1, v/v) gradient by silica gel column chromatography, and then compound **2** was obtained by semi-prep. HPLC (MeOH-H_2_O, 50:50, v/v). Compound **3** (500 mg) was obtained from Fr.8 by repeated recrystallization of CH_2_Cl_2_-MeOH. Fr. 2-2 was separated by silica gel column chromatography, eluted with Petrol-Acetone (6:1, v/v) gradient, recrystallized with petroleum ether to obtain fr.2-2, and then compound **4** (30 mg) were obtained by silica column chromatography.

### Cell Culture

The RAW264.7 cells were cultured in an incubator at 37°C and 5% CO_2_ and maintained in DMEM medium with high glucose containing 10% FBS and 1% double antibiotics (Penicillin and Streptomycin). The cell fusion rate reached about 80%, and the ratio of extended is 1:3. The cells of the logarithmic growth phase were chosen for the following assays.

### Cell Viability Assay

The effects of compounds **1**, **4** on the viability and cytotoxicity of RAW264.7 cells were determined by MTT assay. The method was the same as Wang's experiment (28).

### NO Production Assays

The Griess reaction was applied to determine the NO level secreted by RAW264.7 cells. The cells were treated in a 24-well plate at 1.6 × 10^5^ cells/well for 24 h. Then model group and the experimental group were, respectively, treated with LPS (1 μg/mL), LPS (1 μg/mL) + compounds **1** (50, 25, 12.5 μM), or LPS (1 μg/mL) + compounds **4** (25, 12.5, 6.25 μM) for 24 h, while the control group was treated only with the DMEM medium. After that, the culture supernatant of RAW264.7 cells was collected to test the secretion of nitric oxide according to the Griess reagent kit.

### Measurement of TNF-α and IL-6

RAW264.7 cells were treated according to the above method, and the levels of TNF-α and IL-6 was determined by ELISA kits according to the manufacturer's instructions.

### ROS Level

The content of ROS in RAW264.7 cells was carried by the fluorescent probe DCFH-DA following the kit instructions. The ROS levels was measured using Flow Cytometry.

### Western Blot Analysis

Total protein was obtained after lysing the cells (RIPA lysate). The protein content was determined by BCA protein assay kit. The western blot method was the same as the experiment done by Wang et al. (28).

### Quantitative Real-Time Polymerase Chain Reaction

The process of cell culture and treatment was similar to the above method. Total RNA was isolated from the RAW264.7 cells via Trizol reagent. The method of qRT-PCR was referred to Zang's experiment (29).

### Statistical Analysis

All the experimental data were expressed as mean ± SD. SPSS 19.0 software was used for data statistical analysis. Statistical significance was calculated by the one-way ANOVA analysis. *P* < 0.05 were considered statistically significant.

## Result

### Effects of Compounds 1 and 4 on RAW264.7 Cells Viability

**Figure 2:**
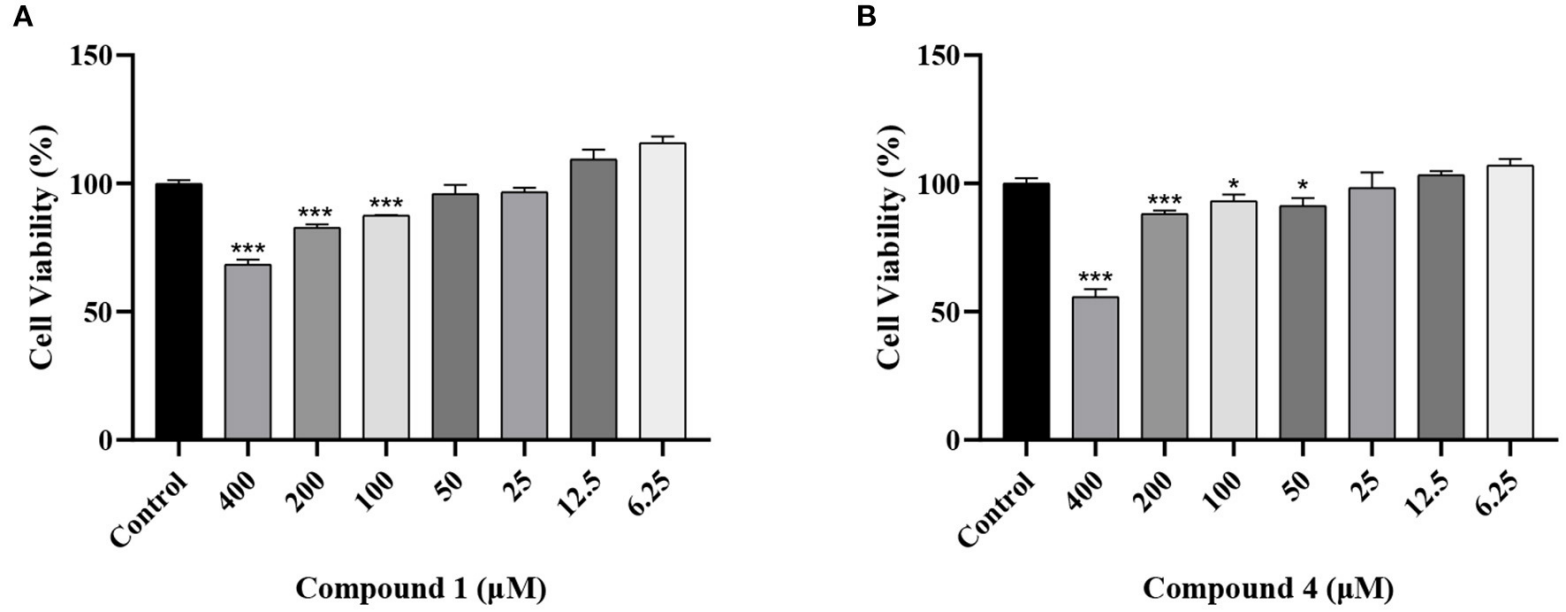
Effects of compound **1 (A)**, compound **4 (B)** on the viability of RAW264.7 cells. The data were expressed as mean ± standard deviation, *n* = 6. ***P < 0.001, *P < 0.05 compared with the control group.

To explore the effects of compounds **1** and **4** on RAW264.7 cells viability, we used MTT assay to test the effects of compound **1** and compound **4** at the dose range of 6.25–400 μM. In Figure 2, compound **1** and compound **4** displayed obvious cytotoxic effects on RAW264.7 cells. Therefore, 50, 25, 12.5 μM of compound **1** and 25, 12.5, 6.25 μM of compound **4** were chosen for the further experiments.

### Effects of Compound 1 and Compound 4 on NO Secretion and mRNA Expression of iNOS in RAW264.7 Cells

**Figure 3:**
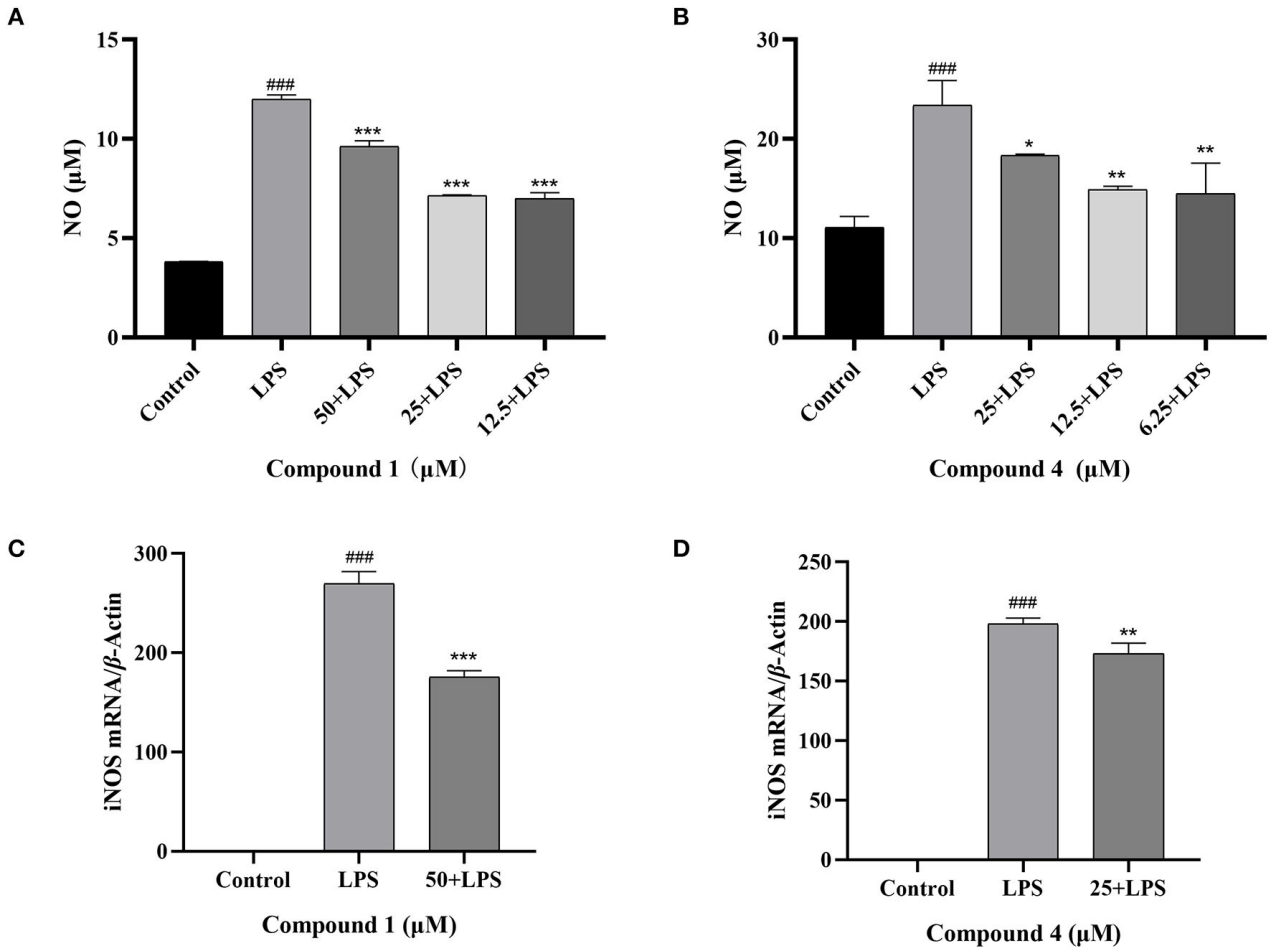
Effects of compound **1 (A)**, compound **4 (B)** on NO production and effects of compound **1 (C)**, compound **4 (D)** on iNOS mRNA expression in RAW264.7 cells. The data were expressed as mean ± standard deviation, *n* = 3. Compared with the control, ###*P* < 0.001. Compared with the LPS group, ***P < 0.001, **P < 0.01, *P < 0.05.

NO is an important inflammatory mediator that played a pivotal role in cell survival and death (30). In Figure 3, the concentration of NO was increased obviously, when RAW264.7 cells were stimulated by LPS. Compound **1** and compound **4** could reduce NO production caused by LPS conspicuously. Under normal physiological conditions, the iNOS activity of cells is almost not expressed. When RAW264.7 cells were induced by LPS, the expression of iNOS in cells increased, resulting in the secretion of NO increased (31). To further verify the way that compound **1** and compound **4** regulated the release of NO, the expression level of iNOS mRNA was detected. The results showed that compound **1** and compound **4** could markedly reduce the expression level of iNOS mRNA in RAW264.7 cells (Figures 3C,D).

### Effects of Compound 1 and Compound 4 on the Secretion of TNF-α, IL-6 in LPS-Induced RAW264.7 Cells

**Figure 4:**
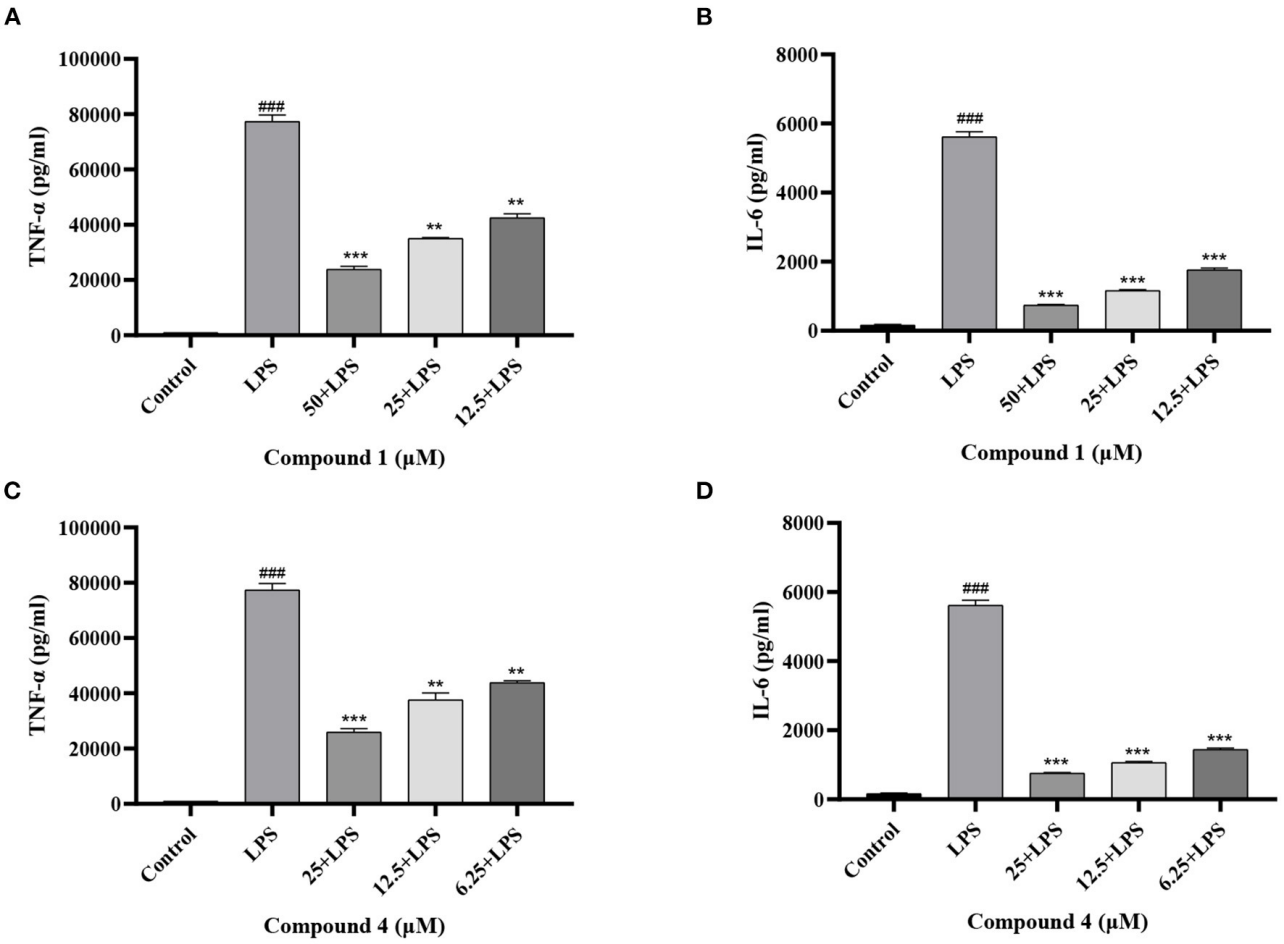
Effects of compound **1 (A,B)** and compound **4 (C,D)** on the secretion of TNF-α, IL-6 in LPS-Induced RAW264.7 Cells. The data were expressed as mean ± standard deviation, *n* = 3. Compared with the control, ^###^*P* < 0.001. Compared with the LPS group, ***P < 0.001, **P < 0.01.

**Figure 5:**
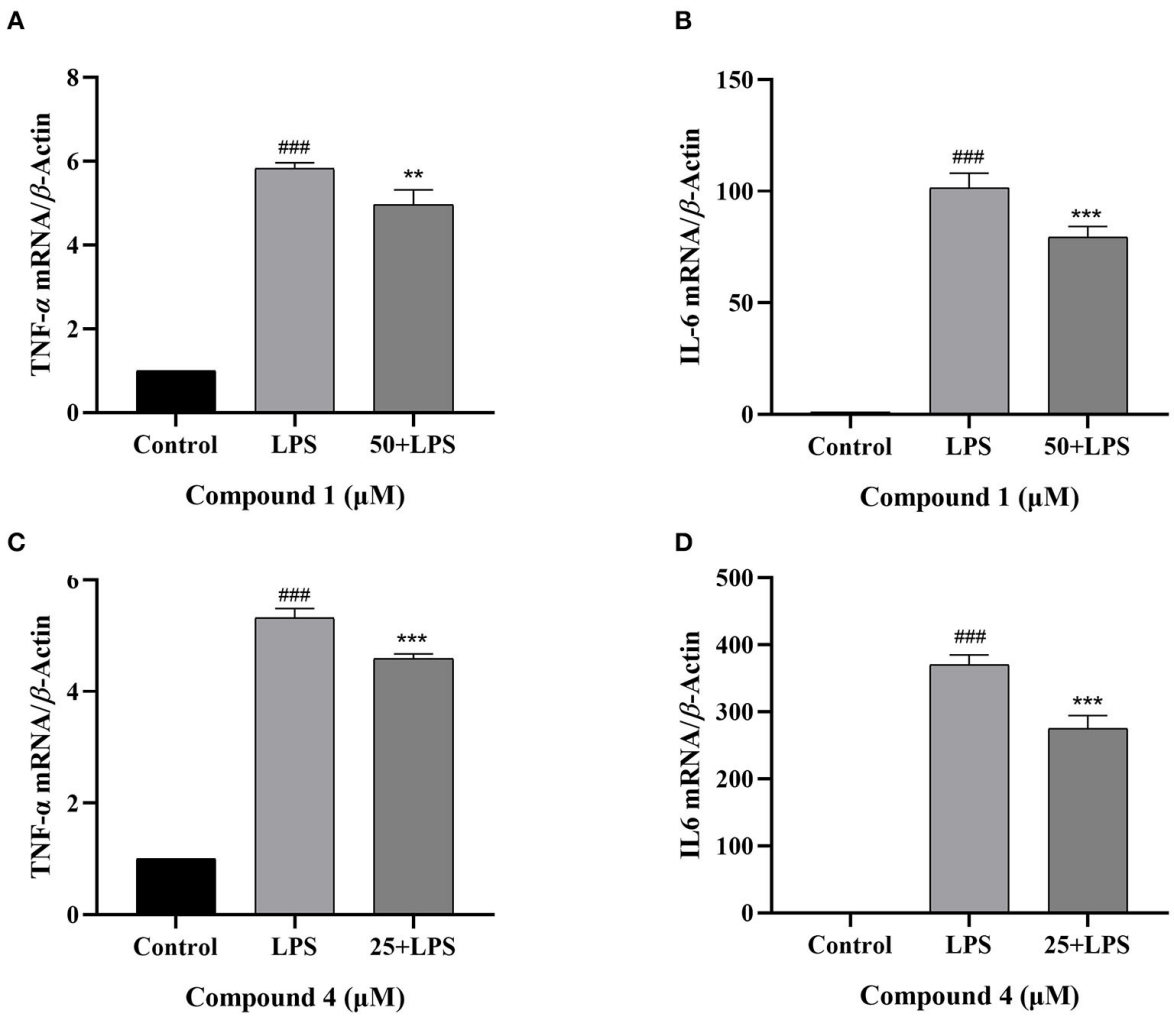
Effects of compound **1 (A,B)** and compound **4 (C,D)** on the expression of TNF-α, IL-6 mRNA in LPS-Induced RAW264.7 cells. The data were expressed as mean ± standard deviation, *n* = 3. Compared with the control, ^###^*P* < 0.001. Compared with the LPS group, ***P < 0.001, **P < 0.01.

TNF-α and IL-6 are mainly produced by macrophages as cytokines with multiple functions (32, 33). In Figure 4, the secretion of TNF-α, IL-6 increased considerably after LPS induction as compared with the control group. Compound **1** and compound **4** both significantly reduced the secretion of TNF-α, IL-6 in a dose dependent manner. Furthermore, the mRNA levels of cytokines were measured by qRT-PCR. As shown in Figure 5, compound **1** (50 μM), compound **4** (25 μM) were found to contribute to a considerable reduction of the increased mRNA expression of TNF-α, IL-6 in the RAW264.7 cells stimulated by LPS.

### Effects of Compound 1 and Compound 4 on LPS-Induced Protein Expression of iNOS and COX-2

**Figure 6:**
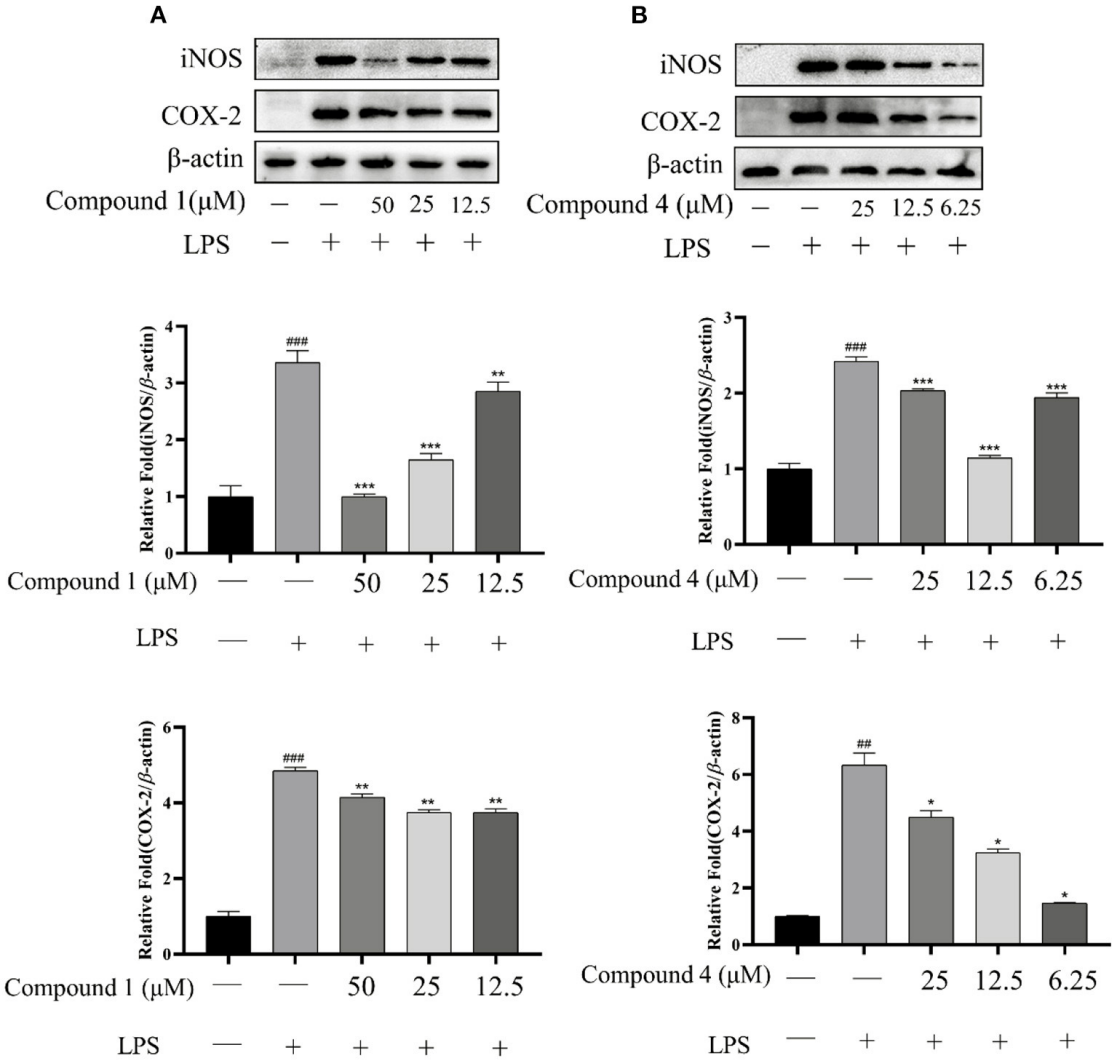
Effects of compound **1 (A)** and compound **4 (B)** on LPS-induced protein expression of iNOS and COX-2. The data were expressed as mean ± standard deviation, *n* = 3. Compared with the control, ^###^*P* < 0.001, ^##^*P* < 0.01. Compared with the LPS group, ***P < 0.001, **P < 0.01, *P < 0.05.

The protein expressions of iNOS and COX-2 were determined to evaluate the effect of compound **1** and compound **4** on proinflammatory mediators. In Figure 6, LPS can significantly increase the expression of iNOS and COX-2 compared with the control group. Meanwhile, the excessive expression of iNOS and COX-2 was inhibited significantly by compound **1** and compound **4** compared with the LPS group.

### Effect of Compound 1 and Compound 4 on LPS-Induced Oxidative Stress

**Figure 7:**
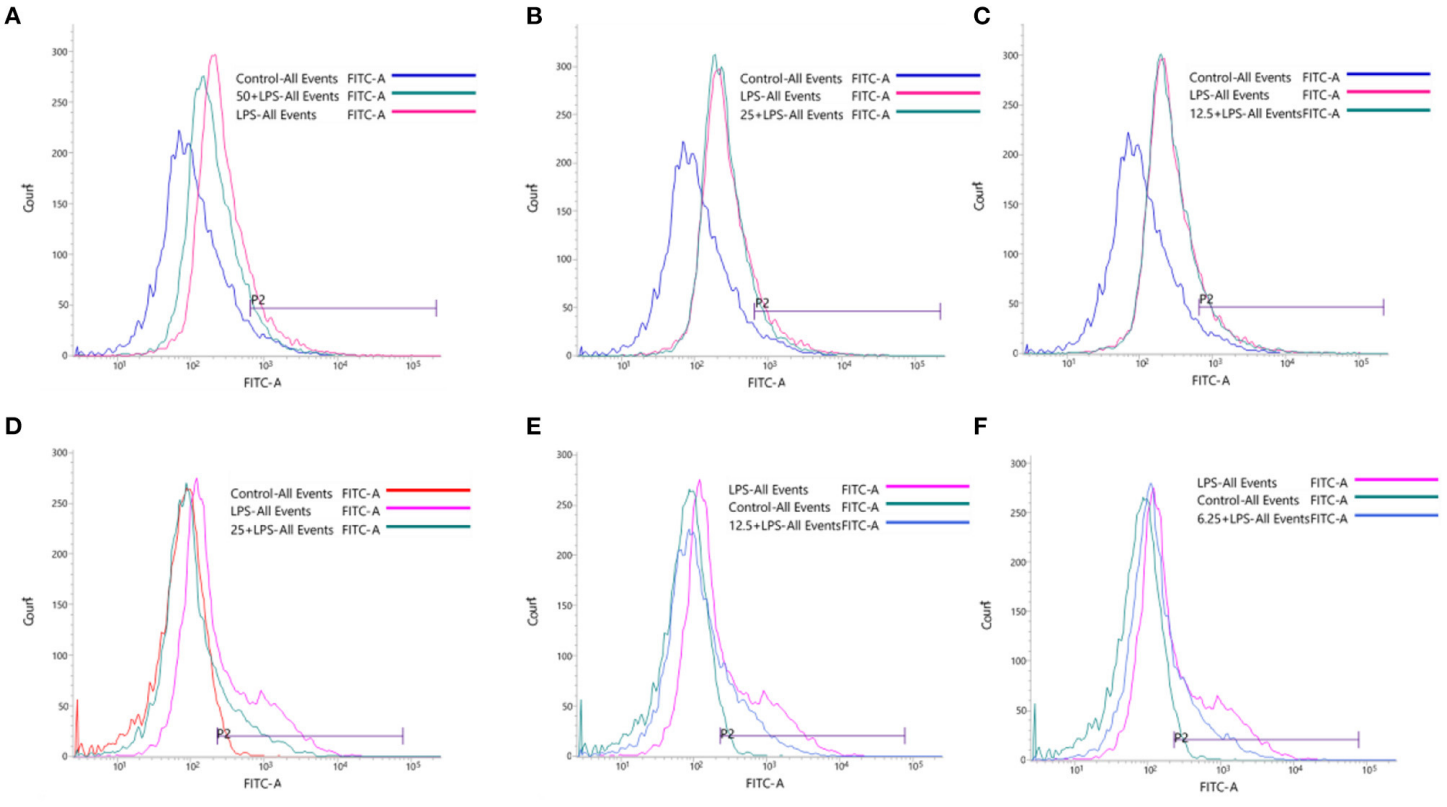
Effects of compound **1** at 50 μM **(A)**, 25 μM **(B)**, 12.5 μM **(C)** and compound **4** at 25 μM **(D)**, 12.5 μM **(E)** and 6.25 μM **(F)** on LPS-induced oxidative stress.

Excessive reactive oxygen species is the major cause of high mortality caused by local inflammation and sepsis (34). In this study, the RAW264.7 cells were stimulated by LPS to release a large number of endogenous ROS, which can mediate the transmission of inflammatory signals in macrophages. In Figure 7, the peak shape of cell fluorescence in the LPS group shifted significantly to the right compared with the control group, which illustrated the intracellular ROS increase sharply. Compound **1** at 50 or 25 μM could effectively inhibit the overexpression of ROS in RAW264.7 cells induced by LPS, while compound **1** at 12.5 μM had little inhibitory effect on it. At the same time, compound **4** at 25, 12.5, and 6.25 μM could markedly inhibit the overexpression of ROS.

### Effects of Compound 1 and Compound 4 on NF-κB Signaling Pathway in RAW264.7 Cells Induced by LPS

**Figure 8:**
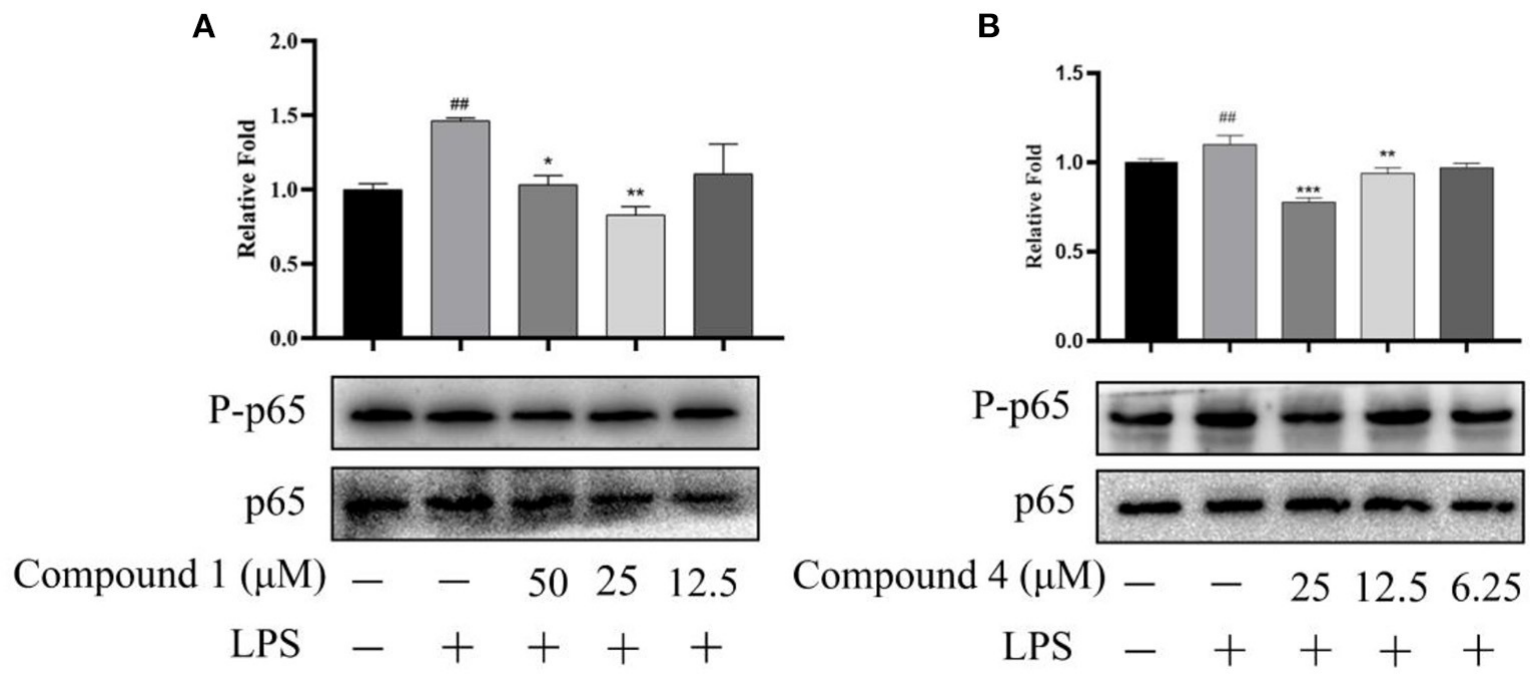
Effects of compound **1 (A)** and compound **4 (B)** on NF-κB signaling pathway. The data were expressed as mean ± standard deviation, *n* = 3. Compared with the control, ^##^*P* < 0.01. Compared with the LPS group, ***P < 0.001, **P < 0.01, *P < 0.05.

When the body has an inflammatory response, multiple signal pathways are activated and coordinated to jointly regulate the expression of pro-inflammatory and anti-inflammatory factors in innate and adaptive immune cells, tissues, or recruited from the blood (35). In Figure 8, compound **1** and compound **4** displayed a significant suppression on the expression of proteins p-p65 in the NF-κB signaling pathways of LPS-induced RAW264.7 cells.

## Discussion

Due to the complexity of different inflammation, developing highly effective and low-toxic drugs is still a challenge for pharmaceutical chemists (36). Alkaloids widely exist in plants and have a variety of anti-inflammatory activities. They can be used as lead compounds or candidate drugs to help us design and find new anti-inflammatory drugs (37). Alkaloids in many natural products are used in the treatment of inflammation. Delavatine A is a special isoquinoline alkaloid isolated from *Incarvillea delavayi*. It was found that it can significantly inhibit the production of proinflammatory mediators (38). A novel steroidal alkaloid, Solanine A, is isolated from *Solanum nigrum* Linn, exhibits significant inhibition of NF-κB, ERK1/2, Akt and STAT1 signaling pathways to exert anti-inflammatory activity in macrophages that induced by LPS/IFNγ (39). Isoquinoline alkaloid glycoside isolated from *Phellodendron chinense Schneid* can inhibit the excessive production of inflammatory mediators and show good anti-inflammatory activity (40). Therefore, exploring alkaloids with therapeutic potential from natural resources is a rich source for discovering and developing new compounds with medical value.

*D. brunonianum* is a folk medicine plant with multiple biological properties, including anti-inflammatory, anti-tumor, anti-viral and anti-oxidation. However, its anti-inflammatory active ingredients and mechanism are unclear that needed to be explored. Studies have shown excessive NO is the most typical biomarker of inflammation (41). And inflammatory cytokines are produced by a variety of cells, the most important of which are macrophages (42). They can play a variety of functions in the process of the inflammatory response, such as activating endothelium and leukocytes and inducing acute phase reactions (43). In this study, we detected the anti-inflammatory activity of four compounds that isolated and identified from *D. brunonianum*. It was found that the alkaloid, compound **1** and compound **4** could significantly reduce the production of pro-inflammatory factors NO, TNF-α, IL-6 caused by LPS, at the same time, this result was verified by PCR at mRNA level. These results indicate that compound **1** and compound **4** have significant anti-inflammatory activity.

iNOS and COX-2 are two important proteins in the initiation and progression of the inflammatory process, and play key roles in the synthesis of NO and PGE2, respectively (44, 45). As an inflammatory mediator, prostaglandin participates in the pathological process of inflammation, cancer, and a variety of cardiovascular diseases (46). In addition, prostaglandin binds to specific receptors to mediate a series of cell activities such as cell proliferation, differentiation, and apoptosis (47). COX is a key enzyme in the process of prostaglandin synthesis (48). Therefore, the expression of iNOS and COX-2 is also overproduction in RAW264.7 cells induced by LPS will be significantly up-regulated. In our studies, the western blot results showed that the excessive expression of iNOS and COX-2 was inhibited significantly by compound **1** and compound **4** compared with the LPS group. This result further shows that compound **1** and compound **4** have anti-inflammatory activity.

Oxidative stress response refers to a state in which the body's highly active molecules such as active oxygen are excessively produced when the body is subjected to various harmful stimuli, and the oxidation and antioxidant effects in the body are out of balance (49). ROS is generally considered to be a harmful inflammatory mediator (50). The production of ROS can promote the release of various inflammatory factors. On the contrary, the increase in the secretion of inflammatory mediators promotes the production of ROS and aggravates the oxidative stress damage of cells (51). In this study, we detected the content of ROS in RAW264.7 stimulated by LPS using Flow Cytometry, the LPS group released a large number of endogenous ROS, and compound **1** and compound **4** could effectively inhibit the overexpression of ROS in LPS-induced RAW264.7 cells, although the effect of the low-dose group of compound **1** is not obvious. Our results further suggest that compound **1** and compound **4** not only have anti-inflammatory activity but also can effectively inhibit the overexpression of ROS.

To further explore the anti-inflammatory mechanism of compound **1** and compound **4**, we detected the expression of key proteins in the NF-κB signaling pathway through western blot experiments. It is well-known that the production of inflammatory cytokines and oxidative stress are related to the activation of NF-κB (35). The NF-κB transcription factor family controls the expression of important regulatory genes in the processes of immunity, inflammation, death and cell proliferation, and is very important as a stressor in the cellular environment (52). In our studies, we detected the expression of key proteins p-p65 and p65, western blot results showed that compound **1** and compound **4** displayed a significant suppression on the expression of key proteins p-p65 in the NF-κB signaling pathways. Thus, we preliminarily speculated that compound **1** and compound **4** exerted the greatest inhibition toward LPS-induced oxidative stress and inflammatory response via the NF-κB signaling pathway. However, how compound **1** and compound **4** regulate the expression of PP65 and activate the NF-κB signaling pathway is still unknown. In the process of inflammation, several different signal pathways may be activated and coordinated with each other to regulate the expression of pro-inflammatory mediators and anti-inflammatory mediators. Therefore, whether compound **1** and compound **4** exert their anti-inflammatory effects through other pathways is also a question worth considering.

## Conclusions

In conclusion, we proved that the alkaloid, extracted and separated from *Delphinium brunonianum* Royle, could reduce the production of pro-inflammatory mediators in LPS-induced RAW264.7 cells, and this effect of compound **1** and compound **4** is involved in the NF-κB signaling pathway. In addition, they can effectively inhibit the overexpression of ROS. Therefore, compound **1** and compound **4** may be promising drugs for the treatment of inflammatory diseases.

## Data Availability Statement

The original contributions presented in the study are included in the article/Supplementary Material, further inquiries can be directed to the corresponding author/s.

## Author Contributions

QT and SC performed experiments and writing original draft preparation. SR and SW analyzed and summarized data. JQ and LW contributed to the data acquisition. CM and LL supervised project administration. WK provided resources, funding, and reviewed the manuscript. All authors read and approved the final paper.

## Funding

This work was funded by Research on Precision Nutrition and Health Food, Department of Science and Technology of Henan Province (CXJD2021006).

## Conflict of Interest

The authors declare that the research was conducted in the absence of any commercial or financial relationships that could be construed as a potential conflict of interest.

## Publisher's Note

All claims expressed in this article are solely those of the authors and do not necessarily represent those of their affiliated organizations, or those of the publisher, the editors and the reviewers. Any product that may be evaluated in this article, or claim that may be made by its manufacturer, is not guaranteed or endorsed by the publisher.
