# Supplementary material for: Two Alkaloids From Delphinium brunonianum Royle, Their Anti-inflammatory and Anti-oxidative Stress Activity via NF-κB Signaling Pathway
Source: Front Nutr. 2022 Jan 20;8:826957. doi: 10.3389/fnut.2021.826957 (PMC8812339; doi:10.3389/fnut.2021.826957)
Supplement: Supplementary file 1 [file Table_1.DOCX]

**Table 1** Primers for RT-PCR

| Gene | Sequences |
| --- | --- |
| iNOS | Forward: 5' - GCTCGCTTTGCCACGGACGA -3'  Reverse: 5' - AAGGCAGCGGGCACATGCAA -3' |
| TNF-*α* | Forward: 5' - CCCTCCTGGCCAACGGCATG -3'  Reverse: 5' - TCGGGGCAGCCTTGTCCCTT -3' |
| IL-6 | Forward: 5' - AGACAAAGCCAGAGTCCTTCAGAGA -3'  Reverse: 5' - GCCACTCCTTCTGTGACTCCAGC -3' |
| β-actin | Forward:5′-TGCTGTCCCTGTATGCCTCT-3′  Reverse:5′-TTTGATGTCACGCACGAT TT-3′ |

**Spectral data for compounds 1-4**

Compound **1**: Delbrunine, light yellow oilines, 95% of purity, has a molecular formula of C_25_H_39_NO_7_. ^1^H-NMR (400 MHz, CDCl_3_) *δ*_H_: 3.79 (d, *J* = 6.2 Hz, 1H, H-1), 1.65 (m, 1H, H-2a), 1.90 (dd, *J* = 16.3Hz, 5.2 Hz, 1H, H-2b), 1.65 (m, 1H, H-3a), 1.44 (m, 1H, H-3b), 3.60 (t, *J* = 5.7 Hz, 1H, H-5), 3.79 (d, *J* = 6.2 Hz, 1H, H-6), 2.08 (m, 1H, H-9), 1.65 (m, 1H, H-10), 1.65 (m, 1H, H-12a), 2.08 (m, 1H, H-12b), 2.39 (d, *J* = 13.6 Hz, 1H, H-13), 4.15 (t, *J* = 5.1 Hz, 1H, H-14), 2.57 (dd, *J* = 16.3Hz, 9.1 Hz, 1H, H-15a), 1.90 (dd, *J* = 16.3Hz, 5.2 Hz, 1H, H-15b), 3.43 (d, *J* = 12.1 Hz, 1H, H-16), 3.09 (d, *J* = 8.9 Hz, 1H, H-17), 3.43 (d, *J* = 12.1 Hz, 1H, H-18a), 3.60 (t, *J* = 5.7 Hz, 1H, H-18b), 2.39 (d, *J* = 13.6 Hz, 1H, H-19a), 2.57 (dd, *J* = 16.3Hz, 9.1 Hz, 1H, H-19b), 2.93, 2.77, (m, 2H, N-CH_2_CH_3_), 1.15 (t, *J* = 7.2 Hz, 3H, N-CH_2_CH_3_), 5.08, 5.12 (s, 2H, OCH_2_O), 3.32 (s, 3H, 6-OCH_3_), 3.34 (s, 3H, 16-OCH_3_), 3.37 (s, 3H, 18-OCH_3_). ^13^C NMR (101 MHz, CDCl_3_) *δ*_C_:71.8 (C-1), 27.3 (C-2), 29.8 (C-3), 37.2 (C-4), 42.1 (C-5), 88.5 (C-6), 92.0 (C-7), 83.5 (C-8), 45.7 (C-9), 46.6 (C-10), 50.7 (C-11), 29.0 (C-12), 38.4 (C-13), 74.8 (C-14), 36.1 (C-15), 81.7 (C-16), 66.1 (C-17), 78.1 (C-18), 57.5 (C-19), 50.4, 13.5 (N-CH_2_-CH_3_), 58.3 (6-OCH_3_), 56.5 (16-OCH_3_), 59.4 (18-OCH_3_), 94.3 (OCH_2_O).

Compound **2**: 4-*O*-*α*-D-Glucosyl benzoic acid, white powder, 98% of purity, has a molecular formula of C_13_H_16_O_8_. ^1^H NMR (400 MHz, CD_3_OD) *δ*_H_ 8.04 (d, *J* = 8.9 Hz, 2H, H-2, 6), 7.22 (m, 2H, H-3,5), 5.50 (d, *J* = 8.0 Hz, 1H, H-1’), 3.58 (m, 1H, H-2’), 3.73 (dd, *J* = 8.0, 3.0 Hz, 1H, H-3’), 4.38 (td, *J* = 10.3, 1.8 Hz, 1H, H-4’), 4.47 (dd, *J* = 11.3, 1.8 Hz, 1H, H-5’), 4.20 (m, 2H, H-6’). ^13^C NMR (101 MHz, CD_3_OD) *δ*_C_ 167.42, 132.6 (C-2, 6), 124.4 (C-1), 117.1 (C-3, 5), 162.5 (C-4), 98.4 (C-1’), 72.9 (C-2’), 73.2 (C-3’), 70.0 (C-4’), 71.8 (C-5’), 66.7 (C-6’).

Compound **3**: Kaempferol 3-*O*-*β*-*D*-glucopyranoside 7-*O*-*α*-*L*-rhamnopyranoside, pale yellow powder, 98% of purity, has a molecular formula of C_27_H_30_O_15_. ^1^H NMR (400 MHz, DMSO) *δ*_H_ 8.08 (d, *J* = 8.9 Hz, 2H, H-2’, 6’), 6.88 (d, *J* = 8.9 Hz, 2H, H-3’, 5’), 6.83 (d, *J* = 2.1 Hz, 1H, H-8), 6.45 (d, *J* = 2.1 Hz, 1H, H-6); Glc: 5.48 (d, *J* = 7.3 Hz, 1H, H-1’’), 3.19 (dd, *J* = 7.7, 14.8 Hz, 2H, H-2’’, 3’’), 3.08 (d, *J* = 5.3 Hz, 2H, H-4’’, 5’’), 3.56 (d, *J* = 11.3 Hz, 2H, H-6’’); Rha: 5.55 (s, 1H, Rha-H-1’’’), 3.84 (s, 1H, H-2’’’), 3.63 (dd, *J* = 9.3, 3.2 Hz, 1H, H-3’’’), 3.30 (m, 1H, H-4’’’), 3.42 (ddd, *J* = 5.47, 11.68, 17.24 Hz, 1H, H-5’’’), 1.11 (d, *J* = 6.1 Hz, 3H, H-6’’’).^13^C NMR (101 MHz, DMSO) *δ*_C_ 155.9 (C-2), 133.4 (C-3), 177.6 (C-4), 156.8 (C-5), 98.4 (C-6), 161.6 (C-7), 94.5 (C-8), 160.9 (C-9), 105.7 (C-10), 120.6 (C-1’), 131.0 (C-2’, 6’), 115.2 (C-3’, 5’), 160.3 (C-4’); Glc: 100.7 (C-1’’), 74.2 (C-2’’), 76.4 (C-3’’), 69.9 (C-4’’), 77.6 (C-5’’), 60.8 (C-6’’); Rha: 99.4 (C-1’’’), 70.1 (C-2’’’), 70.2 (C-3’’’), 71.6 (C-4’’’), 69.8 (C-5’’’), 18.0 (C-6’’’).

Compound **4**: Eldeline, white crystal, 96% of purity, has a molecular formula of C_27_H_41_NO_8_. ^1^H NMR (400 MHz, CDCl_3_) *δ*_H_ 3.48 (m, 1H, H-1), 2.20 (m, 1H, H-2a), 2.08 (m, 2H, H-2b), 1.19 (m, 1H, H-3a), 1.59 (m, 1H, H-3b), 1.56 (s, 1H, H-5), 5.45 (s, 1H, H-6), 3.32 (s, 1H, H-9), 2.49 (m, 1H, H-13), 4.11 (t, *J* = 4.9 Hz, 1H, H-14), 1.81 (dd, *J* = 15.1, 7.2 Hz 1H, H-15a), 2.49 (m,1H, H-15b), 3.18 (t, *J* = 4.9 Hz, 1H, H-16), 3.07 (d, *J* = 2.1 Hz, 1H, H-17), 0.87 (s, 3H, H-18), 2.49 (m,1H, H-19a), 2.75 (m, 1H, H-19b), 2.75 (m, 2H, N-CH_2_CH_3_), 1.05 (t, *J* = 7.2 Hz, 3H, N-CH_2_CH_3_), 4.95, 4.90 (s, 2H, OCH_2_O), 3.25 (s, 3H, 1-OCH_3_), 3.44 (s, 3H, 14-OCH_3_), 3.32 (s, 3H, 16-OCH_3_), 2.08 (s, 3H, COCH_3_), 1.66 (s, 1H, 10-OH). ^13^C NMR (101 MHz, CDCl_3_) *δ*_C_: 79.2 (C-1), 27.1(C-2), 39.4 (C-3), 33.8 (C-4), 50.40 (C-5), 77.4 (C-6), 91.7 (C-7), 84.0 (C-8), 55.5 (C-9), 81.7 (C-10), 50.5 (C-11), 36.6 (C-12), 38.5 (C-13), 81.6 (C-14), 34.8 (C-15), 81.5 (C-16), 63.7 (C-17), 21.9 (C-18), 57.9 (C-19), 50.4, 14.0 (N-CH_2_-CH_3_), 56.0 (1-OCH_3_), 56.9 (14-OCH_3_), 56.4 (16-OCH_3_), 94.0 ( OCH_2_O), 169.9, 25.8 (OCOCH_3_).
